# Supplementary material for: Specific Neural Mechanisms Underlying Humans’ Processing of Information Related to Companion Animals: A Comparison with Domestic Animals and Objects
Source: Animals (Basel). 2025 Oct 31;15(21):3162. doi: 10.3390/ani15213162 (PMC12607319; doi:10.3390/ani15213162)
Supplement: Supplementary file 1 [file animals-15-03162-s001.zip › animals-3833078-supplementary.pdf]

Supplementary Information for

# Specificity of Neural Mechanisms in Companion Animal Information Processing

*Heng Liu, Xinqi Zhou, Jingyuan Lin, Wuji Lin**

*Correspondence to:

Wuji Lin (E-mail: linwuji55555@sicnu.edu.cn)

Supplementary Methods ........ 2
Sample Size Parameters and Estimation ........ 2
Core Parameters for Sample Size Estimation ........ 2
Calculation of Effect Size and Determination of Sample Size ........ 2
Preparation and Evaluation of Experimental Image Materials ........ 3
Initial Image Screening ........ 3
Basis and Rationale for Classification of Animal Stimuli ........ 3
Basis and Rationale for Classification of Object Stimuli ........ 4
Image Valence Evaluation ........ 4
Steps for Data Analysis of Experimental Materials ........ 6
Questionnaire 1 - Pet Attitude Scale (PAS) ........ 6
Questionnaire 2 - Animal Attitude Scale (AAS) ........ 7
Experimental Procedure ........ 8
Practice Phase ........ 9
Formal Experiment Phase ........ 9
Data Acquisition and Analysis ........ 10
FMRI Data Acquisition ........ 10
FMRI Data Preprocessing and Whole-Brain Analysis (GLM) ........ 10
Correlation Analysis ........ 11
gPPI Analysis ........ 13
DCM Analysis ........ 16
Informed Consent Form ........ 18
References ........ 20

# Supplementary Methods

## Sample Size Parameters and Estimation

### Core Parameters for Sample Size Estimation

The sample size calculation in this study is based on the sample size estimation principles for fMRI studies proposed in the literature. Combined with the characteristics of the "cognitive fMRI task in healthy college students", three key parameters were determined:

1. Percentage of signal change ($\mu D$): In the literature, the average $\mu D$ for cognitive tasks (e.g., verbal working memory) is 0.48%. This study refers to this value and sets $\mu D$ = 0.45% - 0.50%.

2. Degree of variation: The literature indicates that after spatial smoothing (a common preprocessing step in fMRI), the average within-subject variance ($\sigma W$) is 0.74% and the average between-subject variance ($\sigma B$) is 0.77%. Since the participants in this study are college students with higher homogeneity, $\sigma W$ is set to 0.70% - 0.75% and $\sigma B$ to 0.65% - 0.75%.

3. Statistical criteria: In accordance with the literature recommendations, the target statistical power is set to 80%, and the conservative significance level is set to $\alpha$=0.002 (two-tailed test, adapted to the requirement of multiple comparison correction in fMRI).

### Calculation of Effect Size and Determination of Sample Size

Using the fMRI effect size formula from the literature, the above parameters were integrated and substituted into the formula for calculation, yielding $\delta \approx 0.68$. Referring to the power curve for $\alpha = 0.002$ in the literature (Figure 9), when $\delta \approx 0.68$ and the statistical power is 80%, 24 to 32 participants are required. Considering that minor interference during scanning may reduce statistical power, the sample size was ultimately expanded to 42 participants to ensure the reliability of the results.

## Preparation and Evaluation of Experimental Image Materials

### Initial Image Screening

The initial images were collected through online searches, with the following selection criteria: 1. Uniform size and background: All images were resized to the same dimensions, and the background was set to pure white to reduce interference during image valence evaluation. 2. Exclusion of distracting elements: Images with overly vibrant colors or excessively unique or exaggerated shapes were removed to ensure consistency and general applicability in emotional induction. 3. Experimental material categories: The materials were divided into four categories: companion animals, neutral animals, positive objects, and neutral objects. For companion animals, 45 images each of cats and dogs were selected. For neutral animals, 45 images each of chickens and ducks were selected. For positive objects, 45 images each of vases and sculptures were selected. For neutral objects, 45 images each of tables and chairs were selected. Additionally, 45 negative images were selected as filler materials and were not presented during the experimental phase.

### Basis and Rationale for Classification of Animal Stimuli

Selection of companion animals (cats, dogs): Based on the general definition of "companion animals", cats and dogs possess a unique social status that transcends that of ordinary livestock (Amiot et al., 2016). Interactions between cats and dogs and humans are typically characterized by intimate emotional bonds (e.g., companionship, emotional comfort) (Martens et al., 2016).

Selection of neutral animals (chickens, ducks): Chickens and ducks were selected as neutral animals primarily based on the weak strength of their emotional bonds with humans and their species-specific roles. Rationale for their neutral emotional valence: Chickens and ducks are primarily perceived as "economic animals" (e.g., providing meat and food ingredients, eggs) or "environmentally associated animals" (e.g., common poultry in rural areas). They are rarely kept as pets, their emotional interactions with humans are dominated by "functional contact", and they lack the unique intimate attachment of companion animals—thereby meeting the neutral criterion of "low emotional relevance".

**Basis and Rationale for Classification of Object Stimuli**

Selection of positive objects (vases, sculptures): Vases and sculptures were selected as positive objects primarily based on their non-utilitarian emotional value and positive cultural associations. Rationale for their positive valence: The core attributes of vases and sculptures are "decorativeness" and "aesthetic quality"—vases are typically used for flower arrangement and decoration, symbolizing an "elegant lifestyle"; sculptures serve as artistic carriers capable of evoking aesthetic pleasure. Their emotional valence primarily derives from "subjective feelings of pleasure".

Selection of neutral objects (tables, chairs): Tables and chairs were selected as neutral objects primarily due to their strong utilitarian function and weak emotional association. Rationale for their neutral valence: Tables and chairs are instrumental items in human daily life, whose core functions include "supporting writing activities and enabling seating". In use, human perception of these objects centers on "whether they meet functional needs" (e.g., height, stability) rather than on emotional experiences.

To summarize, the core "companion attribute" of cats and dogs, the core "economic animal attribute" of chickens and ducks, the core "aesthetic value attribute" of vases and sculptures, and the core "practical value attribute" of tables and chairs are highly consistent with their respective species-specific or object-specific roles, thereby ensuring the reliability of valence assessments for the selected stimuli.

**Image Valence Evaluation**

To confirm that there were no valence differences between positive animal and objects images, as well as between neutral animal and objects images, and to ensure significant differences between positive and neutral images, a valence evaluation was performed on the 360 collected images. The specific steps were as follows: 1. Participants: 30 individuals were recruited for the evaluation, all of whom were native Mandarin speakers without a history of major psychological or mental disorders. 2. Evaluation Procedure: The images were presented using Presentation 0.71 software. Participants viewed each image individually on a laptop and rated its valence on a

7-point scale (1 = extremely negative, 7 = extremely positive). 3. Evaluation Control: Participants rated each image as it was presented, with no time constraints. Completing the evaluation of all images took approximately 15-20 minutes per participant. 4. Instructions: Participants were informed that they would see a series of images and rate each on a 1-7 scale, where 1 indicated extreme negativity, 7 indicated extreme positivity, and 4 indicated neutrality. They were also informed that there were no right or wrong answers. After evaluation, 48 images from each condition were retained as formal experimental materials, including 24 images of cats and dogs, 24 of chickens and ducks, 24 of vases and sculptures, and 24 of tables and chairs. Additionally, 6 images from each of the four categories (3 of cats and dogs, chickens and ducks, vases and sculptures, tables and chairs) were selected for practice trials, which were not included in the formal experiment (see Supplementary Figure 1).

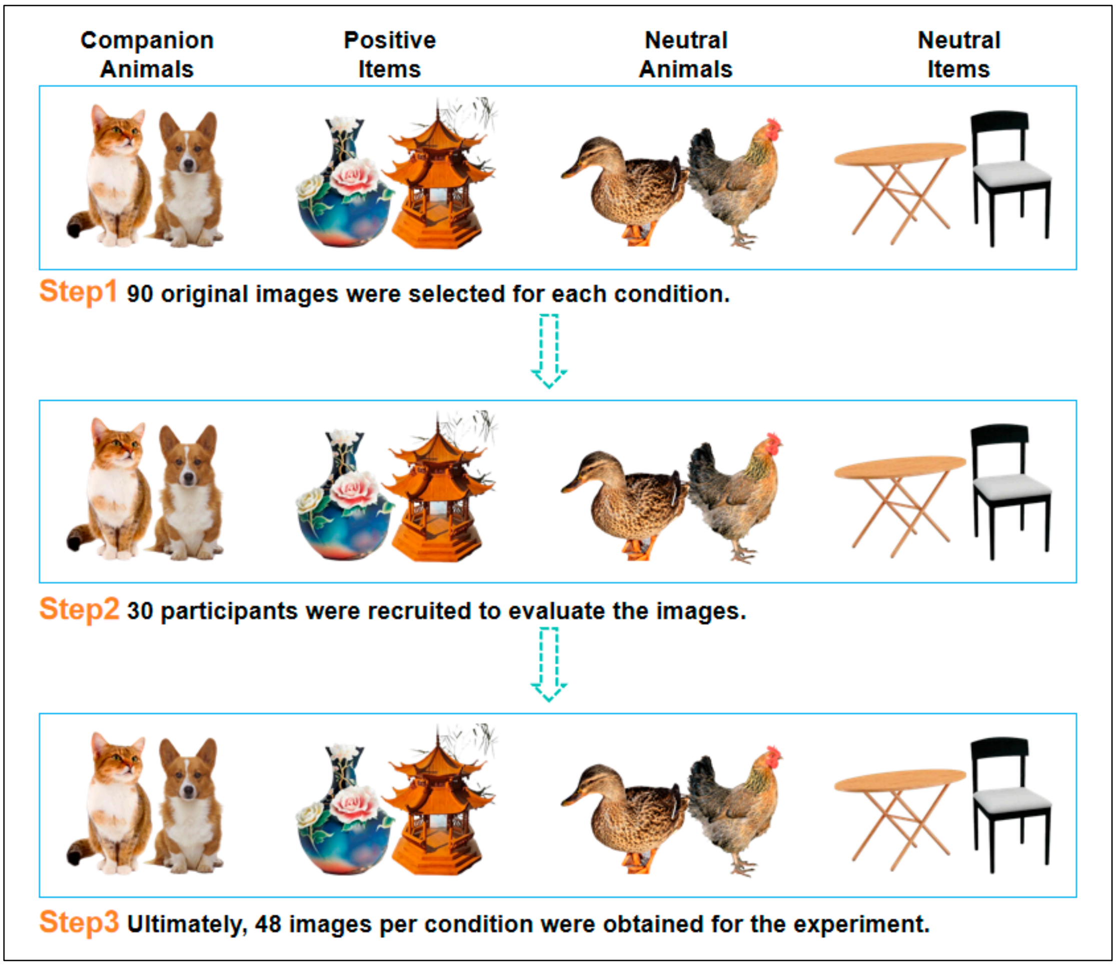


**Supplementary Figure 1: Procedure for the Preparation and Evaluation of Experimental Materials**

**Steps for Data Analysis of Experimental Materials**

1. Valence Comparison: Analyzed the valence differences between image categories. The main focus was to confirm that there was no significant valence difference between positive animal and positive objects images, as well as between neutral animal and neutral objects images. 2. Significance Test: An independent samples t-test was performed to verify a significant valence difference between positive and neutral images ($p < 0.05$). 3. Image Selection: A total of 54 images per category, meeting the experimental design requirements, were retained. Of these, 6 images per category were used for practice trials, and the remaining 48 were used in the formal experiment.

**Questionnaire 1 - Pet Attitude Scale (PAS)**

The Pet Attitude Scale (PAS) consists of 18 items rated on a 7-point Likert scale, with response options ranging from Strongly Disagree to Strongly Agree. A higher PAS score indicates a more positive attitude towards pets, with items 4, 6, 9, 12, 13, 15, and 17 being reverse scored. The questions include:

1. I really enjoy seeing pets enjoy their food (if I have one).

2. My pet is more important to me than any friend (if I have one).

3. I want to keep a pet in my home.

*4. I think keeping pets is a waste of money.

5. Having a pet adds joy to my life (if I have one).

*6. I think pets should always be kept outside.

7. I spend time playing with my pet every day (if I have one).

8. I occasionally communicate with my pet to understand what it wants to express (if I have one).

*9. The world would be a better place if people stopped spending so much time caring for their pets and started caring more for others.

10. I like hand-feeding my pet (if I have one).

11. I like pets.

*12. Pets should be in the wild or in zoos, not in homes.

*13. Keeping pets in the house would cause a lot of damage to furniture.

14. I like household pets.

*15. Pets are fun, but not worth having because they are too much trouble.

16. I often talk to my pet (if I have one).

*17. I hate pets.

18. You should treat pets like members of your family.

**Questionnaire 2 - Animal Attitude Scale (AAS)**

The original version included 29 items, designed by Herzog, Betchart, and Pittman in 1991. Items 21 to 29 formed a subscale intended to assess people's tendencies to help animals. Factor analysis showed that all items loaded onto a single factor, indicating they measured the same concept. Therefore, researchers concluded that items 21 to 29 did not independently assess the tendency to help animals and decided to remove them. Items marked with "*" are scored in reverse. Responses are rated on a 5-point scale ranging from 1 (Strongly Disagree) to 5 (Strongly Agree). Higher scores reflect a more positive attitude toward animals.

Below are 20 statements regarding the use of animals. Please indicate your level of agreement for each: (Strongly Disagree, Disagree, Uncertain, Agree, Strongly Agree)

1. Hunting wild animals purely for entertainment is morally wrong.

*2. There is nothing wrong with using animals in medical research.

3. Extremely severe punishment, including imprisonment, should be imposed on people who participate in cockfighting.

4. Wild animals, such as minks and raccoons, should not be caught to make coats from their fur.

*5. There is nothing morally wrong with hunting wild animals for food.

*6. I think people who oppose raising animals for meat are overly emotional.

7. Many animal-related scientific studies are unnecessary and cruel.

*8. I think raising cattle and pigs for human consumption is perfectly acceptable.

*9. Humans have the right to use animals as we wish.

10. Whaling and dolphin hunting should be stopped immediately, even if it means some people will lose their jobs.

11. I sometimes feel uneasy when I see animals confined in zoo cages.

*12. Overall, I think human economic interests are more important than preserving more habitats for wildlife.

*13. I think too much emphasis is placed on animal welfare nowadays, and we should focus more on addressing human needs.

*14. It is reasonable to raise animals for their fur.

*15. Some biological knowledge can only be learned by dissecting existing animals, such as dissecting cats.

*16. If we want to conquer cancer, heart disease, and AIDS, we must continue to use animals in related research.

17. Millions of dogs are euthanized in animal shelters each year, so breeding purebred dogs for the pet market is unethical.

*18. It is reasonable to raise animals under crowded conditions to produce cheap meat, eggs, and dairy products.

19. Using animals (such as rabbits) to test the safety of cosmetics and household products is unnecessary and should be stopped.

20. Using animals in competitive shows and circuses is cruel.

## Experimental Procedure

Upon arrival at the experimental site, participants completed an MRI screening form, informed consent form, and questionnaires, followed by a practice session and then the formal experiment (see Supplementary Figure 2).

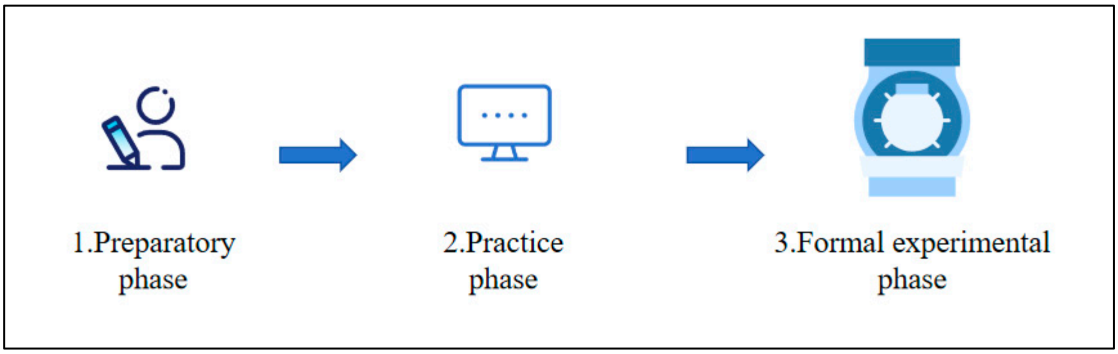


**Supplementary Figure 2: Experimental Procedure**

### Practice Phase

Before the formal experiment, participants practiced outside the MRI chamber. The practice session was identical to the formal experiment except for the number of trials, designed to familiarize participants with the procedure. Once they were fully familiar with the experiment, participants entered the chamber for the formal session.

### Formal Experiment Phase

The formal experiment included two sessions, each with 12 blocks. As shown in Supplementary Figure 3, key prompts were presented based on the upcoming block's content. For example, if the upcoming block was "Positive Animals (cats, dogs)," "cat" appeared on the left and "dog" on the right, prompting participants to press the left key for "cat" and the right for "dog" (key presses were used to ensure attentive image processing; they were not analyzed separately). Other blocks were designed similarly. Stimuli in each block were presented in an "image-fixation-image" sequence against a black background. Images were displayed for 3000 milliseconds, and fixation points for 1000 milliseconds. A longer fixation point was presented between blocks, with a randomly set duration of 5 to 16 seconds. Participants were asked if they needed a break between sessions.

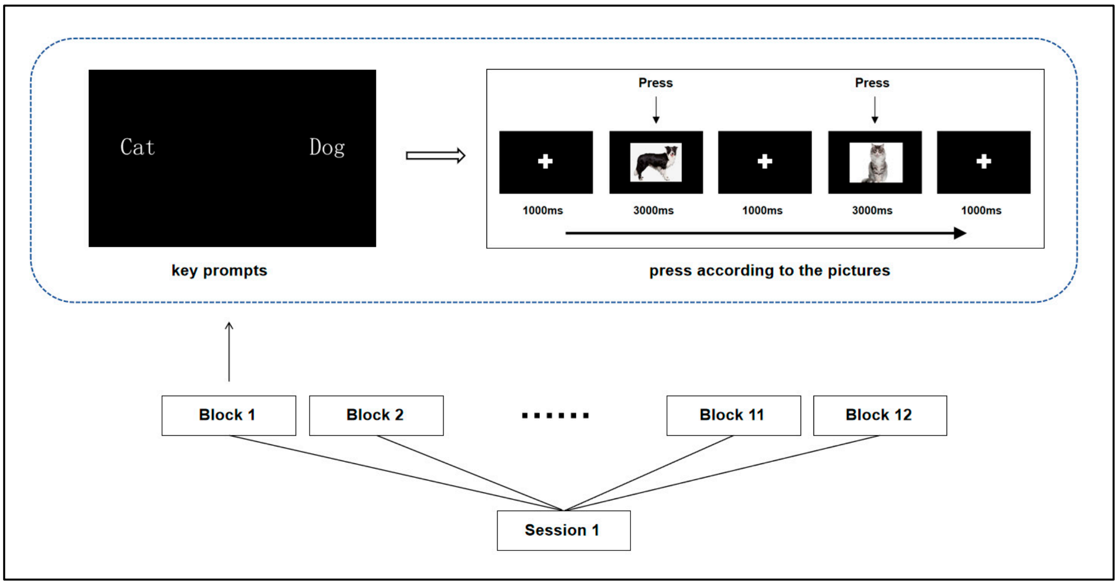


**Supplementary Figure 3: Experimental Task**

## Data Acquisition and Analysis

### FMRI Data Acquisition

Data were collected using a Siemens 3T MRI scanner (Siemens, Erlangen, Germany) with a 64-channel head-neck coil. Structural images were acquired using T1-weighted 3D MPRAGE with the following parameters: Repetition time (TR) = 2530 ms, Echo time (TE) = 2.34 ms, slice thickness = 0.9 mm, 192 slices, matrix size = 256×256, Field of view (FoV) = 220 mm, flip angle = 7°, and voxel size = 0.9×0.9×0.9 $mm^3$. Functional images were acquired using T2-weighted echo-planar imaging in an interleaved order with the following parameters: TR = 2000 ms, TE = 30 ms, FoV = 224×224 $mm^2$, flip angle = 90°, matrix size = 224×224, slice gap = 0.3 mm, voxel size = 2×2×2 $mm^3$, acquiring 62 transverse slices of 2 mm thickness covering the whole brain. In the MRI scanner, participants viewed the screen through a mirror on top of the head coil and responded to stimuli using a keypad attached to their right hand. The keypad had two keys, labeled 1 and 3, corresponding to left and right directions. Before scanning, participants were given soundproof earplugs, and sponges were placed on either side of their head near the coil base to minimize head movement. During structural imaging, participants were instructed to relax, while during functional imaging, they were asked to keep their eyes open and stay focused.

### FMRI Data Preprocessing and Whole-Brain Analysis (GLM)

Preprocessing, individual-level analysis, and group-level analysis of functional magnetic resonance imaging (fMRI) data were conducted using the MATLAB toolboxes SPM 12 (Wellcome Trust Center for Neuroimaging, London, UK, http://www.fil.ion.ucl.ac.uk/spm/software/spm12) and DPARSF 5.2. DPARSF is a MATLAB toolbox developed based on Statistical Parametric Mapping (SPM) and functions from the fMRI data analysis toolbox, designed for fMRI pipeline analysis. After organizing the DICOM files and setting parameters, DPARSF provides all preprocessed data. DPARSF can also generate a report to exclude pa

rticipants with excessive head motion and produce images to evaluate normalization quality. Additionally, DPARSF can extract time series from regions of interest (Yan et al., 2010; Yan et al., 2016).

Task-based fMRI data were preprocessed using DPARSF 5.2 on the MATLAB R2018b platform: (1) T1DICOM to NIFTI and EPIDICOM to NIFTI: Convert T1 and EPI DICOM files to NIFTI format. (2) Slice Timing Correction: To correct for differences in acquisition times across slices and ensure that all slices in a TR are synchronized. (3) Realignment: Correct for head movement to align images from different time points of the same participant, as involuntary movements could obscure the hemodynamic response. Participants with translational movement >3 mm or rotational movement >3° were excluded. (4) Normalization: Normalize each participant's brain to a standard space to minimize errors due to inter-individual differences in brain size and shape. Specifically, fMRI images were normalized using the EPI template in SPM. (5) Detrending: Correct for linear drift caused by factors like scanner temperature changes or participant adaptation. (6) Remove Confounding Factors: Regress out head motion, global signal, and cerebrospinal fluid (CSF) signal. (7) Smoothing: Apply a Gaussian kernel with an FWHM of 6 mm to the fMRI data.

After preprocessing, individual-level analysis was performed in SPM12 using a General Linear Model (GLM). First-level GLM analysis included separate regressors for companion animals, positive objects, and neutral animals, with contrasts for the six head motion parameters. Group-level analysis was performed in DPARSF 5.2 using t-tests to determine specific activation effects of companion animal processing at the whole-brain level. Multiple comparisons were corrected using Gaussian Random-Field (GRF) theory, with significance thresholds of $p < 0.01$ at the voxel level, $p < 0.05$ at the cluster level, two-tailed test, and cluster size > 30 voxels.

**Correlation Analysis**

Correlation analysis was performed using the Correlation Analysis toolbox in DPARSF 5.2 to evaluate the relationship between companion animal-specific

activation from whole-brain analysis and scores on the Pet Attitude Scale (PAS) and Animal Attitude Scale (AAS). No significant effects were found (Supplementary Figure 4A). Given the nonsignificant results and prior research indicating an association between companion animal ownership and cognitive performance as well as neural activity (McDonough et al., 2022; Hayama et al., 2016), we conducted separate correlation analyses for pet owners (PO) and non-pet owners (NPO), relating their specific activation results to PAS and AAS scores. Analysis showed a positive correlation between activation in the right inferior parietal lobule of pet owners and PAS scores, but it was not significant after correction (Supplementary Figure 4B). The BOLD signal percentage extracted from the right inferior parietal lobule mask (using MarsBaR) was correlated with PAS scores in SPSS, showing a positive relationship. No significant correlation was found for non-pet owners in any brain region (Supplementary Figure 4C). Brain regions showing a positive correlation with PAS scores in the pet owner group ($p < 0.01$) were saved as brain masks, and BOLD signal values were extracted and imported into SPSS to quantify the correlation between neural activity and individual attitudes.

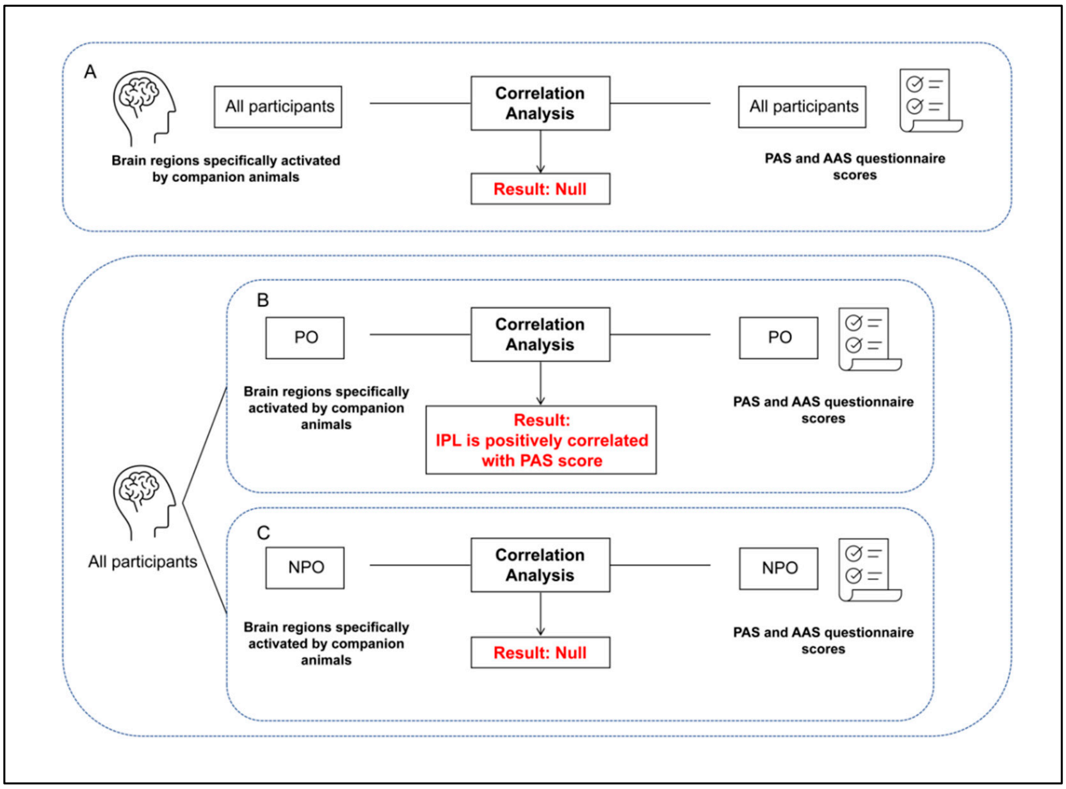


**Supplementary Figure 4: Correlation Analysis**

**gPPI Analysis**

To investigate whether changes in connectivity between brain regions are related to processing companion animal information, we conducted Generalized PsychoPhysiological Interactions (gPPI) analysis (McLaren et al., 2012). The analysis code is available at https://doi.org/10.17605/OSF.IO/YADXN. The companion animal-specific activation regions (right IPL, right MOG, left SFG, and left PCu) were saved as masks to serve as seed points for whole-brain functional connectivity analysis (Supplementary Figure 5). The average time series of the seed points was deconvolved to reveal neuronal activity (physiological variables) and multiplied by the vector of each experimental condition (psychological variables) to form psychophysiological interaction vectors. The interaction vector was convolved with the standard HRF to create the gPPI regressor for the region of interest. After individual-level analysis, group-level functional connectivity analysis was conducted using SPM12 to identify brain regions with significant connectivity to the seed points under the companion animal condition. It should be noted that although the gPPI results ($p < 0.05$ and cluster size > 50) were not corrected for strict multiple comparisons, which may increase false positive risk, strict correction may also miss real experimental effects (Han et al., 2018). To control false positives while avoiding missing real effects, we saved the uncorrected brain regions as masks and extracted BOLD signal values to calculate functional connectivity strength with the seed points, identifying regions (left CC, right ACC, left IPL, left PCu) with significantly higher connectivity under the "companion animal" condition compared to others (see Supplementary Table 1 for details). This suggests that our final results are more likely to represent true neurophysiological phenomena rather than statistical noise or other artifacts.

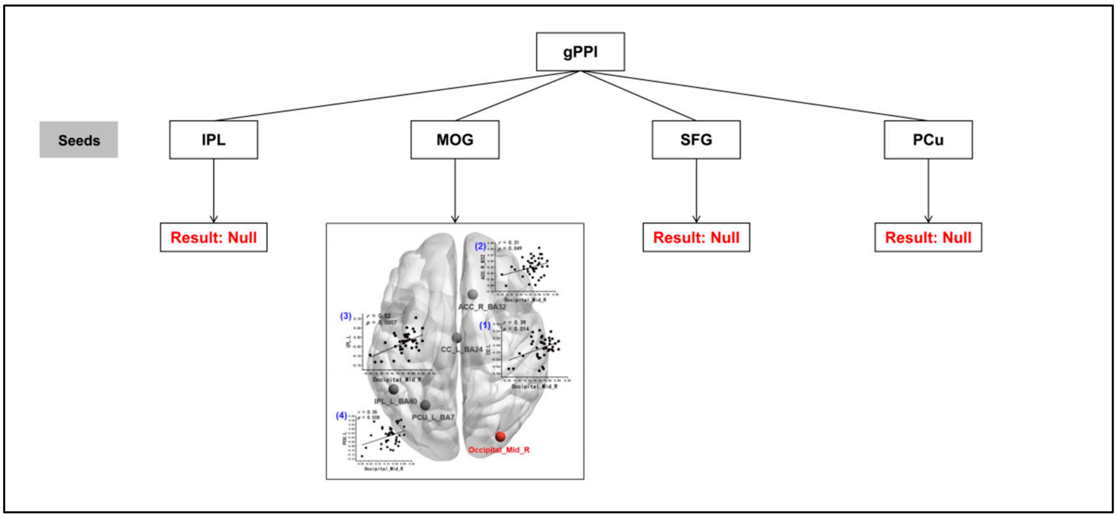


**Supplementary Figure 5: gPPI Analysis Results Using Activated Brain Regions as Seed Points**

| | Seed: R.MOG | | | | | | | | | | | | | | | |
|---|---|---|---|---|---|---|---|---|---|---|---|---|---|---|---|---|
| | L.CC | | R.ACC | | L.IPL | | L.PCu | | R.SPL | | R.FuG | | L.MFG | | R.IPL | |
| | *r* | *p* | *r* | *p* | *r* | *p* | *r* | *p* | *r* | *p* | *r* | *p* | *r* | *p* | *r* | *p* |
| Condition1 | .385* | .014 | .314* | .049 | .515** | <.001 | .347* | .028 | .531** | <.001 | -.133 | .412 | .099 | .543 | .204 | .207 |
| Condition2 | .082 | .616 | .211 | .192 | -.008 | .963 | -.128 | .43 | .568** | <.001 | .166 | .305 | .052 | .748 | .207 | .199 |
| Condition3 | .236 | .143 | .249 | .122 | .261 | .104 | .334* | .035 | .455** | .003 | -.022 | .891 | .422** | .007 | -.102 | .53 |
| Condition4 | .03 | .854 | .216 | .182 | .074 | .649 | .104 | .522 | .502** | .001 | -.012 | .939 | .492** | <.001 | .189 | .244 |

**Supplementary Table 1:** gPPI Analysis Results Using R.MOG as the Seed Point. Condition1 = Companion Animals, Condition2 = Positive objects, Condition3 = Neutral Animals, Condition4 = Neutral objects. Abbreviations: R = Right, L = Left; MOG = Middle Occipital Gyrus; CC = Cingulate Cortex; ACC = Anterior Cingulate Cortex; IPL = Inferior Parietal Lobule; PCu = Precuneus; SPL = Superior Parietal Lobule; FuG = Fusiform Gyrus; MFG = Middle Frontal Gyrus.

**DCM Analysis**

To further investigate how companion animals modulate connectivity between brain regions, we used fMRI Parametric Empirical Bayes (PEB) Dynamic Causal Modeling (DCM) to estimate effective connectivity within and between regions (Friston et al., 2003). The detailed procedure is shown in Supplementary Figure 6. The analysis code is available at https://doi.org/10.17605/OSF.IO/YADXN.

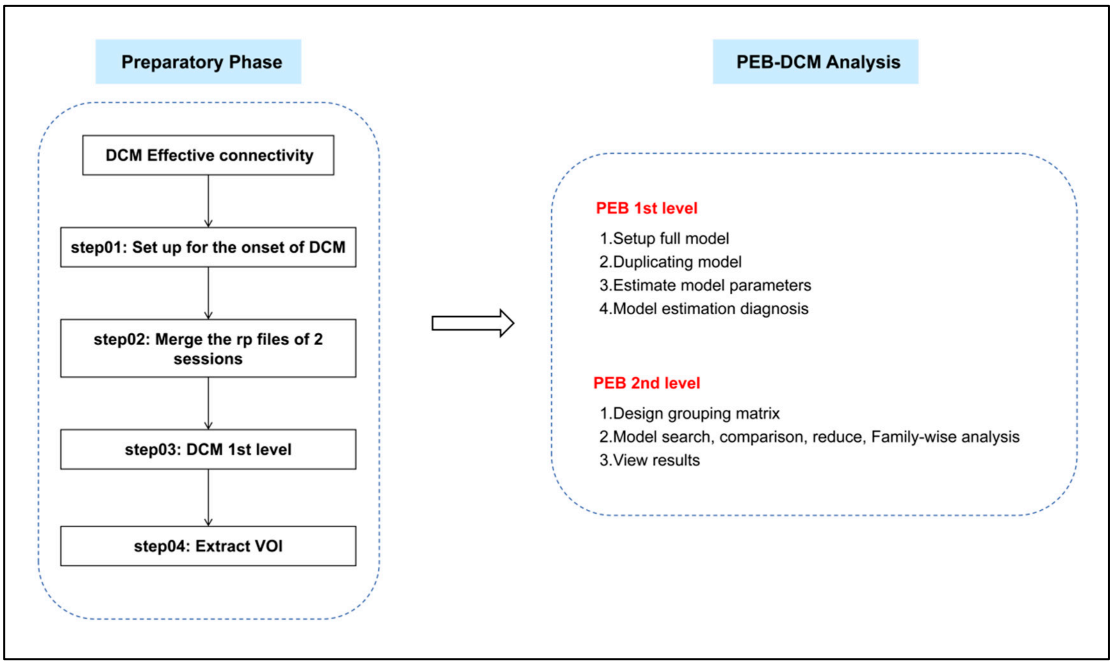


**Supplementary Figure 6: Analysis Procedure**

Because of the specific principles of DCM, the model matrix used in GLM activation analysis cannot be used directly, requiring the construction of a new matrix for Dynamic Causal Modeling (Stephan, 2010; Zeidman et al., 2019).

In PEB-DCM (Parametric Empirical Bayes - Dynamic Causal Modeling) analysis, the A, B, and C matrices represent different connections and modulatory effects in the brain network. These matrices are core components of DCM, describing causal relationships and modulatory mechanisms between brain regions. A matrix (Intrinsic Connections): Represents intrinsic or "background" connectivity between brain regions in the absence of external stimuli or task conditions. B matrix (Modulatory Connections): Describes how experimental conditions change connectivity between regions, indicating whether certain connections are strengthened or weakened under specific conditions. C matrix (Driving Inputs): Describes how

external stimuli directly drive or activate specific brain regions without involving inter-regional connectivity (Supplementary Figure 7). To explore the impact of companion animals, all stimuli (all trials) were used as driving inputs, while companion animals served as modulatory inputs for matrix reconstruction (Supplementary Figure 8).

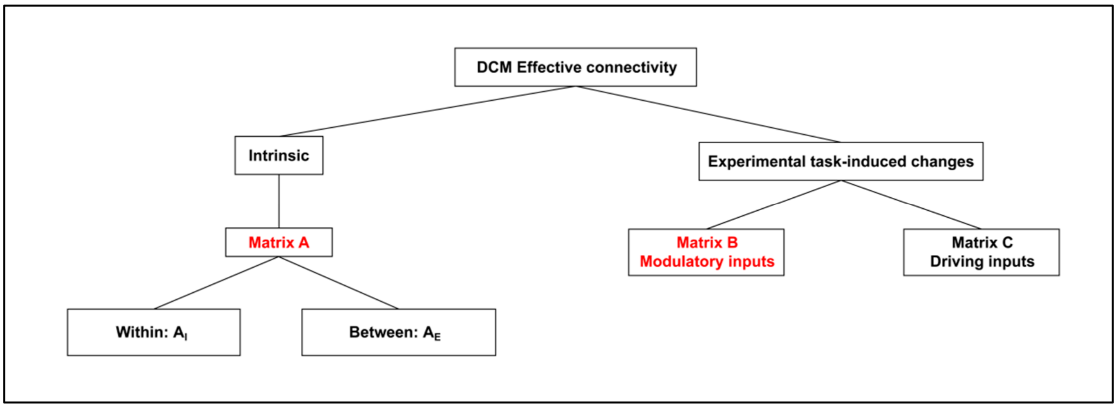


**Supplementary Figure 7: DCM Matrix Composition**

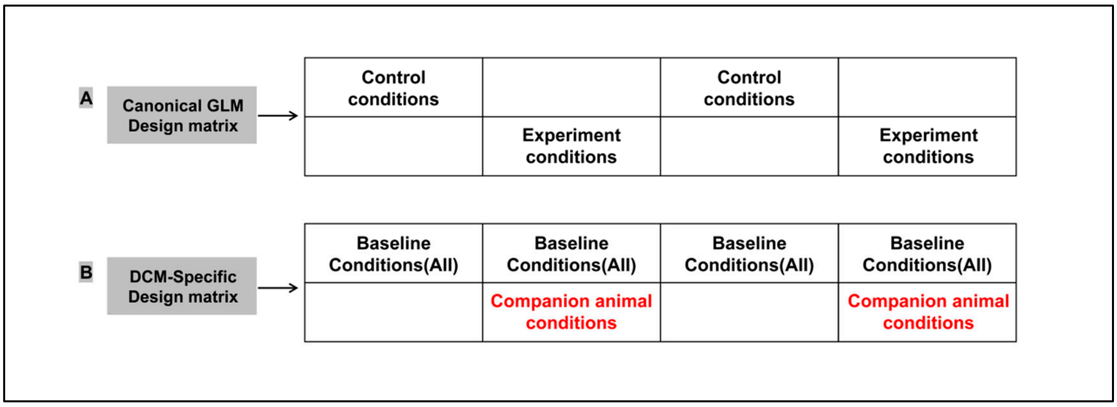


**Supplementary Figure 8: DCM Matrix Reconstruction**

The regions of interest (ROIs) in the DCM analysis were selected based on gPPI results (MOG, CC, ACC, IPL, PCu). By parameterizing the regressors for all relevant brain regions, the A matrix was established based on intrinsic connectivity, the B matrix on the neural response to companion animal processing (modulatory input), and the C matrix on the neural response to all experimental stimuli (driving input).

Using Laplace transformation (Friston et al., 2007), we first constructed a complete dynamic causal model (full model) under the PEB framework for one participant's data, then replicated it for other participants to obtain individual models. The first complete dynamic causal model (full model) provided a reference for

estimating expected connection strength, posterior probability, covariance, and iteratively constructing competing models (reduced models). Bayesian modeling was used to estimate parameters for all competing models (Friston et al., 2016).

After performing first-level individual analysis and model construction, we used the Parametric Empirical Bayes (PEB) method to determine changes in connectivity strength at the second-level group analysis (Friston et al., 2016). Unlike traditional estimation methods, the Bayesian model identifies commonalities and differences in connectivity strength at the group level, considering both the mean and variance of individual-level parameter estimates. This means that individuals with more precise parameter estimates have a greater influence on group-level estimation, while those with greater uncertainty have a reduced influence (Dietz et al., 2020; Zeidman et al., 2019). The advantage of PEB is that it provides both the posterior distribution of connectivity strength at the group level and the model's marginal likelihood for comparison (Penny, 2012; Rigoux et al., 2014). Finally, Bayesian linear regression was used to compare PEB models, determining effective connectivity between regions of interest during companion animal processing for all participants and assessing differences in connectivity between pet owners and non-pet owners.

There are two approaches for handling multiple sessions in the experimental task: (1) concatenating all sessions for combined analysis (Zeidman et al., 2019), or (2) analyzing each session individually and averaging for group analysis (Song et al., 2021). Since DCM can only handle one session at a time, we chose to merge the two sessions for analysis. Specifically, the design matrices of each session were concatenated to construct a design matrix suitable for DCM, and the head movement files were concatenated to create a head movement file for DCM.

# Informed Consent Form

**Research Institution:** Sichuan Normal University

**Principal Investigators:** Heng Liu, Wuji Lin

**Dear Participants:**

We invite you to participate in a research project approved by the Social Science Fund of the Ministry of Education. This study will be conducted at the Institute of Brain and Psychological Sciences. This study has been reviewed and approved by the Ethics Review Committee of the Institute of Brain and Psychological Sciences of Sichuan Normal University.

**1. What is the duration of the experiment?**

The total duration of the experiment is approximately 50-60 minutes.

**2. What are the eligibility criteria for participating in this study?**

Normal vision or corrected-to-normal vision; no major illnesses, head trauma, claustrophobia, or neurological disorders; no recent surgeries, and no metal implants or medical devices in the body; no fear of images of "cats, dogs, chickens, or ducks".

**3. What are the risks of participating in this study?**

All potential risks have been disclosed, and the researchers conducting the experiment will strictly follow the instrument's operational standards.

**Meanwhile, as a participant, you have the following rights regarding this experiment:**

**1. The right to voluntarily decide whether to continue participating in the experiment**

During participation, regardless of whether any harm occurs (or the severity of such harm), you may notify the researchers at any time to request withdrawal from the study. Your data will not be included in the research results, and your rights will not be affected as a result.

**2. Compensation**

If you participate in this study, you will receive a remuneration of 50 yuan in recognition of your contribution to this research. Furthermore, if you suffer harm due to your participation in the study, you may contact us at any time.

**Privacy Matters:** If you decide to participate in this study, all research team members will strictly keep confidential your personal information related to your participation in the experiment and your data collected during the experiment. Your files will be stored in locked file cabinets, and only researchers will have access to them. When the results of this study are published, no personal information of yours will be disclosed.

Participant's Statement: I have read the above introduction regarding this study. The

researchers have fully explained and elaborated on the purpose, procedures of this study, as well as the potential risks and benefits associated with participating in this study, and have answered all my relevant questions. I voluntarily participate in this study.

**Signature:**

**Date:**

## References


Amiot, C., Bastian, B., & Martens, P. (2016). People and companion animals: It takes two to tango. BioScience, 66(7), 552-560. DOI 10.1093/BIOSCI/BIW051

Desmond, J. E., & Glover, G. H. (2002). Estimating sample size in functional MRI (fMRI) neuroimaging studies: statistical power analyses. Journal of neuroscience methods, 118(2), 115-128.

Dietz, M. J., Zhou, Y., Veddum, L., Frith, C. D., & Bliksted, V. F. (2020). Aberrant effective connectivity is associated with positive symptoms in first-episode schizophrenia. NeuroImage: Clinical, 28, 102444.

Friston K J, Harrison L, Penny W. Dynamic causal modelling[J]. Neuroimage, 2003, 19(4): 1273-1302.

Friston K J, Litvak V, Oswal A, et al. Bayesian model reduction and empirical Bayes for group (DCM) studies[J]. Neuroimage, 2016, 128: 413-431.

Friston K, Mattout J, Trujillo-Barreto N, et al. Variational free energy and the Laplace approximation[J]. Neuroimage, 2007, 34(1): 220-234.

Han H, Glenn A L. Evaluating methods of correcting for multiple comparisons implemented in SPM12 in social neuroscience fMRI studies: an example from moral psychology[J]. Social neuroscience, 2018, 13(3): 257-267.

Hayama S, Chang L, Gumus K, et al. Neural correlates for perception of companion animal photographs[J]. Neuropsychologia, 2016, 85: 278-286.

Martens, P., Enders-Slegers, M. J., & Walker, J. K. (2016). The emotional lives of companion animals: Attachment and subjective claims by owners of cats and dogs. Anthrozoös, 29(1), 73-88.DOI 10.1080/08927936.2015.1075299

McDonough I M, Erwin H B, Sin N L, et al. Pet ownership is associated with greater cognitive and brain health in a cross-sectional sample across the adult lifespan[J]. Frontiers in aging neuroscience, 2022, 14: 953889.

McLaren D G, Ries M L, Xu G, et al. A generalized form of context-dependent psychophysiological interactions (gPPI): a comparison to standard approaches[J]. Neuroimage, 2012, 61(4): 1277-1286.

Penny W D. Comparing dynamic causal models using AIC, BIC and free energy[J]. Neuroimage, 2012, 59(1): 319-330.

Rigoux L, Stephan K E, Friston K J, et al. Bayesian model selection for group studies—revisited[J]. Neuroimage, 2014, 84: 971-985.

Song Y, Su Q, Yang Q, et al. Feedforward and feedback pathways of nociceptive and tactile processing in human somatosensory system: a study of dynamic causal modeling of fMRI data[J]. NeuroImage, 2021, 234: 117957.

Stephan K E, Friston K J. Analyzing effective connectivity with functional magnetic resonance imaging[J]. Wiley Interdisciplinary Reviews: Cognitive Science, 2010, 1(3): 446-459.

Yan C G, Wang X D, Zuo X N, et al. DPABI: data processing & analysis for (resting-state) brain imaging[J]. Neuroinformatics, 2016, 14: 339-351.

Yan C, Zang Y. DPARSF: a MATLAB toolbox for" pipeline" data analysis of resting-state fMRI[J]. Frontiers in systems neuroscience, 2010, 4: 1377.

Zeidman P, Jafarian A, Corbin N, et al. A guide to group effective connectivity analysis, part 1: First level analysis with DCM for fMRI[J]. Neuroimage, 2019, 200: 174-190.

Zeidman P, Jafarian A, Seghier M L, et al. A guide to group effective connectivity analysis, part 2: Second level analysis with PEB[J]. Neuroimage, 2019, 200: 12-25.
